# Virulent Epidemics and Scope of Healthcare Workers' Duty of Care

**DOI:** 10.3201/eid1208.060360

**Published:** 2006-08

**Authors:** Daniel K. Sokol

**Affiliations:** * Imperial College, London, United Kingdom

**Keywords:** duty of care, avian influenza, bioethics, SARS, policy review

## Abstract

The phrase "duty of care" is, at best, too vague and, at worst, ethically dangerous. The nature and scope of the duty need to be determined, and conflicting duties must be recognized and acknowledged. Duty of care is neither fixed nor absolute but heavily dependent on context. The normal risk level of the working environment, the healthcare worker's specialty, the likely harm and benefits of treatment, and the competing obligations deriving from the worker's multiple roles will all influence the limits of the duty of care. As experts anticipate the arrival of an avian influenza pandemic in humans, discussion of this matter is urgently needed.

Epidemiologists are warning against an impending pandemic of avian influenza that could kill several million people (*1*). This possibility raises an urgent and thorny ethical question: Are healthcare professionals obligated to care for patients during virulent epidemics of infectious disease?

## Duty of Care

Duty of care, in the medical context, is often invoked as a sort of quasi-biblical commandment, akin to "do not lie" or "do not murder." In a document submitted to the Severe Acute Respiratory Syndrome (SARS) Expert Panel Secretariat, Godkin and Markwell suggest that policy guidelines on the duty of care (which they term duty *to* care) should state that healthcare professionals' duty to care extends to a public health emergency in outbreak conditions (*2*). The authors however suggest that healthcare employers have a set of reciprocal responsibilities toward their staffs, which include duties to inform, protect, and support healthcare personnel. Singer et al., in an article on the ethical issues raised by SARS in Toronto, briefly discuss the duty to care before concluding that the 9 authors "could not reach consensus on the issue of duty of care, particularly regarding the extent to which healthcare workers are obligated to risk their lives in delivering clinical care" (*3*). The term "duty of care" (which I take to be synonymous with duty to care) is, at best, too vague and, at worst, ethically dangerous. For these reasons, the phrase should be modified in favor of more specific descriptions of the obligations of healthcare workers.

## Special Obligation of Doctors to Benefit Their Patients

By virtue of their profession, doctors and nurses have more stringent obligations of beneficence than most. They have obligations to a specified group of persons (their patients) that nonmedical personnel have no obligation to help. The term "duty of care" refers to these special obligations. In its bare form, however, the phrase gives no indication of the precise nature of the duty, nor of its limits. Its definitional vagueness, combined with its rhetorical appeal, may be used to justify actions without the need for rational deliberation. During the SARS outbreaks in Toronto, the phrase was often used as a self-standing argument for active involvement on the part of medical staff, without any critical examination of its meaning. Used in this manner, the term may become a subtle instrument of intimidation, pressuring healthcare workers into working in circumstances that they consider morally, psychologically, or physically unacceptable. The phrase duty of care can thus be ethically dangerous by giving the illusion of legitimate moral justification.

To be of any use, the phrase needs to be fleshed out. Are there limits to the duty? Should doctors do everything in their power to benefit their patients? The answer, surely, is no. Doctors are under no moral obligation to donate one of their kidneys to one of their patients, for example. They may, of course, choose to do so, but their act would exceed the demands of everyday morality. What distinguishes normal duty from acting beyond the call of duty, however, is not always clear-cut; the boundary between the 2 categories is fuzzy (*4*).

### Contingency of the Limits of Duty of Care

Defining the limits of the duty of care is a daunting task, strewn with philosophical and logistical difficulties. As the example of the kidney-giving doctors shows, the duty is not absolute but, rather, constrained by several factors. First, the limits of the duty should be a function of the normal risk level. A doctor practicing in Kinshasa, Democratic Republic of Congo (DRC), for instance, is going to incur more risk than a doctor in rural Dorset, England. The diseases are many and the facilities few in DRC. Every nurse or doctor, by accepting a post, is usually aware of the perils of treating infected patients. The appearance of an exotic, highly virulent disease, however, challenges healthcare workers to question their interpretation of the duty of care, in particular, its limits. This challenge was apparent both in the HIV/AIDS epidemics of the 1980s in the United States and in the 2003 SARS outbreaks in Toronto, in which doctors and nurses refused to treat afflicted patients on the grounds that they presented too great a danger (*2**,**5*). This phenomenon is also likely to occur if the anticipated avian influenza epidemic affects Western hospitals. In light of these historical precedents, hospitals may want to inform prospective staff members of what is expected in crisis situations before, rather than in the midst of, an emergency. By using comparisons and statistics, hospitals could indicate the sorts of risks healthcare staff are expected to handle.

Another factor in defining acceptable risk levels relates to the healthcare worker's specialty. Within the same hospital, an emergency care physician, as a first responder to many critically ill or injured persons, is obviously more at risk than, for example, a dermatologist. By entering into a specialty, doctors implicitly consent to a range of risks and responsibilities associated with the job. The outer limit of acceptable personal risk will fall further along the continuum of risk for some specialists (e.g., infectious disease physicians) than for others (e.g., dermatologists or rheumatologists). During the SARS outbreaks in Toronto, the persons most at risk were nurses and infectious diseases (ID) specialists. As a result of their specialist training, they may have felt a stronger obligation to participate than doctors in other areas of medicine.

## Doctors as Multiple Agents

Doctors, although they belong to their own professional community and adhere to its set of rules, are also part of the broader community and therefore subject to the same rights and duties as other members. The 2 spheres of obligation, professional and personal, are both separate and overlapping. They are separate in that the obligations of doctors toward their patients give them rights that nonmedical members of the society do not possess, such as opening someone's abdomen to remove an appendix. The spheres are overlapping, however, in that their role as doctors does not completely absolve their responsibilities as members of the broader community. The immunity from sanction is specific, not general. A gynecologist may legitimately examine intimate parts of his or her patient but cannot drive beyond the speed limit or steal apples from the market stall. With the acquisition of additional duties and rights conferred by the profession, the doctor also agrees to relinquish certain rights enjoyed by others. By entering into the profession, a doctor agrees not only to abide by new rules but also to accept dangers that would be unacceptable to many (e.g., performing a delicate, invasive procedure on a patient with hepatitis or HIV/AIDS).

In times of crisis, the duties deriving from doctors' multiple roles may come into conflict. Doctors, for instance, may have a duty to care for their SARS or avian influenza–infected patients as well as a duty to care for their own children by protecting them (and hence themselves) from infection. So a further problem with the duty to care, aside from its vagueness, is that it fails to consider the holder of the duty as a multiple agent belonging to a broader community. Doctors, in such situations, play several incompatible roles—doctor, spouse, parent, for example—and they must deal with them as best they can. The limits of the duty of care are thus also defined by the strengths of competing rights and duties.

## Virtues of Patients and Their Duty of Care

Whereas much has been written on what makes a good doctor, scant attention has been devoted to the good patient (*6**,**7*). Pellegrino and Thomasma, in For the Patient's Good, devote a chapter to the "good patient" (*8*). "Patients," they write, "must relate to physicians in all of the virtuous ways that govern human interrelationships and social conduct" (*8*). The authors identify 4 key virtues for the good patient: truthfulness, compliance, tolerance, and trust. The virtue most pertinent to this discussion is tolerance. In their examination of tolerance, Pellegrino and Thomasma mention the patients' need to understand the limitations and fallibility of medicine and to care for the well-being of their fellow patients (*8*).

The virtue of tolerance should also require patients to acknowledge healthcare workers' plurality of roles, as well as their fears and concerns in the face of severe risk. If these fears are well founded and reach such a level that medical staff are worried for their life or that of their loved ones, the virtuous patient ought to allow them to step down from their role as caregivers. In such cases, insisting that they continue in this role would reflect a lack of compassion and understanding. Patients should be entitled to ask for a replacement who is less anxious or prone to panic, but they cannot force other persons to undergo extreme stress against their wishes.

When a physician visited the 1995 Ebola virus outbreak in Kikwit (DRC), he found 30 dying patients in an abandoned hospital, left to care for themselves amid rotting corpses, sometimes in the same bed (*9*). Was the last doctor justified in leaving the patients, or should he or she have been obliged to single-handedly treat the highly and dangerously infectious Ebola patients? The answer depends, at least in part, on the actual risk to the doctor and the potential benefits (including the alleviation of pain and distress) that his or her presence will bring to the patients. If the actual risk for serious illness or death for the doctor is low and the benefits of treatment substantial, then he or she may have an obligation to remain. If, however, the lack of protective equipment means that the chances of infection are high and no, or trivially small, benefits will result for the patients (as is often the case with Ebola), then the doctor may justifiably abandon the doomed patients. Virtuous patients, aware of the high risk and the futility of treatment, would not force a doctor to care for them in such circumstances. Patients too have a duty to care for healthcare workers. Part of this duty is not to require doctors to transcend the bounds of reasonable risk during treatment and to respect and acknowledge their roles outside the realm of medicine.

As potential participants in the drama and as holders of a duty of care toward healthcare workers, the general public also should be involved in setting limits to duty. Some form of dialogue between the public and the medical profession, through the media, public consultations, and educational establishments, could help establish a mutually acceptable set of limits.

## Impact on Patient Trust

The justified abandonment of patients by doctors arguably will result in the harm or even death of these patients. Moreover, public trust in doctors will diminish as persons realize that they, like the 30 forsaken Ebola patients at Kikwit General Hospital, might be left on their own as soon as the risk reaches a certain level. The patients at Kikwit died alone, abandoned by both medical staff and their own frightened relatives. So tragic is the situation that it seems counterintuitive to justify the actions of the nurses and doctors. Yet, before passing judgment, comparing this situation with another hypothetical situation may be useful.

If a swimmer in an isolated but supervised beach starts to drown 50 meters from the shore, the lifeguard may reasonably be expected to attempt a rescue. This, after all, is the lifeguard's duty as a qualified professional. If, however, the person is drowning 2 miles out and is surrounded by a school of hungry, man-eating sharks, then one cannot expect the solitary lifeguard to dive among the sharks to save the swimmer, even if that means the swimmer will certainly die and even if the lifeguard has a small chance of saving him or her (at great personal risk).

The lifeguard cannot be criticized for not interfering, even though his or her prima facie duty is to rescue drowning persons. Likewise, the fact that doctors can, in exceptional circumstances, refuse to treat patients does not necessarily entail a moral wrong, no matter how serious the consequences to the abandoned patients. As long as patients hold realistic expectations of the limits of doctors' duty of care, no trust should be lost when these limits are transgressed.

## Urgent Need

In the last 20 years, various outbreaks of severe infectious diseases, from Ebola virus infection to SARS, have highlighted the need for a more precise account of the duties and obligations of healthcare professionals. The impending avian influenza epidemic makes such an account urgent. The concept of duty of care, in its bare form, is too vague to be helpful. Its limits are not fixed, but contingent on various factors, from the working environment's normal risk level to the healthcare worker's specialty and the range of other obligations that derive from his or her multiple roles. To clarify this overlooked topic, empirical social science research should be conducted to illuminate the views and reasoning of physicians, patients, and members of the public on the limits of the duty of care. Philosophical reflection on the issue as well would do much to clarify this overlooked topic. As dramatic as it may sound, delineating the limits of the duty of care may prevent large numbers of doctors from abandoning their patients in a crisis. Such abandonment has happened in the past and may occur again.

In light of the potentially catastrophic impact of avian influenza on human health and economic well-being, this topic should engender a burst of activity and debate in hospitals, universities, and medical journals. We should explore not only the nebulous limits of the duty of care but also infection control measures, staff training and involvement, the role of medical students and volunteers, the triaging of incoming patients, and the logistics of treatment, depending on the severity of the epidemic, as well as the lessons learned from past epidemics. However difficult the task, these issues should best be tackled now, in times of relative calm, rather than in times of pandemic turbulence.
